# A clinical study on clinical features,manifestations and drug resistance of melioidosis

**DOI:** 10.12669/pjms.38.8.5506

**Published:** 2022

**Authors:** Huang Hui, Yan Sheng, Xiaosheng Han, Suimei Wang, Gang Zhang, Xiaobing Wei

**Affiliations:** 1 Huang Hui, Department of Clinical Laboratory, Haikou People’s Hospital, Affiliated Haikou Hospital of Xiangya Medical College, Central South University, Haikou 570208, Hainan, China; 2 Yan Sheng, Department of Clinical Laboratory, Haikou People’s Hospital, Affiliated Haikou Hospital of Xiangya Medical College, Central South University, Haikou 570208, Hainan, China; 3 Xiaosheng Han, Department of Clinical Laboratory, Haikou People’s Hospital, Affiliated Haikou Hospital of Xiangya Medical College, Central South University, Haikou 570208, Hainan, China; 4 Suimei Wang, Department of Clinical Laboratory, Haikou People’s Hospital, Affiliated Haikou Hospital of Xiangya Medical College, Central South University, Haikou 570208, Hainan, China; 5 Gang Zhang, General Surgery, Affiliated Haikou Hospital of Xiangya Medical College, Central South University, Haikou 570208, Hainan, China; 6 Xiaobing Wei, Department of Clinical Laboratory, Haikou People’s Hospital, Affiliated Haikou Hospital of Xiangya Medical College, Central South University, Haikou 570208, Hainan, China

**Keywords:** Melioidosis, Clinical features, *Burkholderia pseudomallei*, Drug resistance, Antibiotic

## Abstract

**Objectives:**

To explore the clinical features, manifestations and drug resistance of melioidosis.

**Methods:**

The clinical data of 45 melioidosis patients treated by Affiliated Haikou Hospital of Xiangya Medical College, Central South University during January 2015 and January 2020 were studied. Informaiton collected included age, birthplace, area of residence, sex, ethnic group, clinical symptoms or signs, colony culture results, degree of drug resistance, treatment regimens and outcomes, department of initial diagnosis, and specimen type, thereby giving an analysis of the clinical features, manifestations, and treatment outcomes of the disease. Body fluids were obtained from all patients to analyze the drug resistance of the bacterium *Burkholderia pseudomallei* based on colony culture and susceptibility test results.

**Results:**

In the 45 cases of melioidosis, the clinical manifestations predominantly included low fever (31.11%), pulmonary infection (22.22%); auxiliary examinations often suggested increases in C reactive protein (CRP) (73.33%), white blood cell count (#WBC) (68.89%) and procalcitonin (PCT) (66.67%); susceptibility test results showed that Burkholderia pseudomallei yielded high sensitive rate to antibacterials including trimethoprim–sulfamethoxazole (SXT) (88.89%),ceftazidime (CAZ)(93.33%), meropenem (MEM) (100.00%), imipenem (IPM)(100.00%); Of the 45 melioidosis patients, 23 were cured (51.11%), and 21 showed an improvement (33.33%) or remained stable (13.33%); A relatively high percentage of the patients experienced post-discharge recurrence/aggravation (recurrence within six months: 4.44%; recurrence within a year: 6.67%)

**Conclusions:**

Low fever, pulmonary infection, and increases in serum inflammatory markers are major clinical features of melioidosis. *Burkholderia pseudomallei* presents high resistance rates to antibacterials such as GEN, FEP, AMP, and IPM.

## INTRODUCTION

Melioidosis is an infectious disease pernicious to humans and animals. Clinically, patients with melioidosis often have such symptoms as malaise, low fever, phymata, chest distress and so on. 1,2 With *Burkholderia pseudomallei* being the direct cause of the disease, melioidosis is likely to be overlooked or misdiagnosed because of its obscure symptoms that resemble those of common diseases such as common cold and pneumonia.^3^ Melioidosis predominantly occurs in tropical climates, with *Burkholderia pseudomallei* affecting humans and animals exposed to water, air, and soil containing the bacterium.^4^,^5^ In Hainan Province, melioidosis is an endemic disease that threatens the local population’s health. However, the clinical diagnosis and treatment of melioidosis still have enormous room for development because there is a lack of clinical studies on the epidemiology o melioidosis and the drug resistance of *Burkholderia pseudomallei* to antibiotics. To reduce the morbidity of the disease, it is essential to implement pertinent prevention and control measures based on a thorough analysis of its clinical features and manifestations and the drug resistance of *Burkholderia pseudomallei*. Therefore, this study explored the clinical features and manifestations of melioidosis and investigated the drug resistance of *Burkholderia pseudomallei*.

## METHODS

The medical records of 45 melioidosis patients treated by Affiliated Haikou Hospital of Xiangya Medical College,Central South University between January 2015 and January 2020 were used for the analysis of epidemiological characteristics of melioidosis. All medical records were derived from the hospital’s Inpatient Information Management System and imported into Excel 2019 for an accuracy check and descriptive analysis.

### Inclusion criteria:

- Confirmed to have *Burkholderia pseudomallei* infection according to colony cultures^6^ using body fluid specimens (blood, pleural fluid, sputum, pus, urine, secretions, etc.);
- Administered with antibiotics and necessary treatment by Haikou Hospital of Xiangya School of Medicine, Central South University following the susceptibility test;

Having complete, explicit medical records (age, birthplace, area of residence, sex, ethnic group, colony culture results, clinical symptoms or signs, degree of drug resistance, treatment regimens and outcomes, department of initial diagnosis, and specimen type, etc.). Since the susceptibility test results might be affected by antibiotics, patients who were given antibiotic therapy during the last 2 months were excluded.

### Ethical Approval:

The study was approved by the Institutional Ethics Committee of Affiliated Haikou Hospital of Xiangya Medical College,Central South University at October 11, 2021(No.:202016 ), and written informed consent was obtained from all participants.

### Basic information collection:

Demographic features (age, birthplace, area of residence, sex, ethnic group) and clinical features (clinical symptoms or signs, colony culture results, degree of drug resistance, treatment regimens and outcomes, department of initial diagnosis, specimen type) were sorted out for further data processing. Descriptive statistics were applied to the medical records based on the inclusion and exclusion criteria via Excel 2019 to analyze their clinical features and manifestations.

### Instruments and reagents:

VITEK 2 automated ID/AST instrument (Biomerieux, France); WalkAway 40 automated microbial identification culture flask (Siemens, Germany); BX51 fluorescence microscope (Olympus, Japan); Avanti high-speed centrifuge (Beckman Coulter, USA); Attune NxT flow cytometer (Thermo Fisher Scientific, USA); thermostatic freezer (Siemens, Germany); slides, centrifuge tubes and other consumables (Wuhan Huicheng Biotechnology Co., Ltd., China); RPMI-1640 media (Anhui Tiankang Medical Technology Co., Ltd., China).

### Culture and susceptibility analysis:

5% sheep blood agarwere applied to in vitro culture of bacterial isolates. VITEK 2 automated ID/AST instrument (Biomerieux, France) was used for microbial identification and antibiotic susceptibility testing, and the results were interpreted according to the Performance Standards for Antimicrobial Susceptibility Testing (27th Edition) issued by the Clinical and Laboratory Standards Institute (CLSI M100-S27).7 Antibiotic susceptibility testing involved trimethoprim–sulfamethoxazole (SXT), ciprofloxacin (CIP),, meropenem (MEM), ceftazidime (CAZ), imipenem (IPM).

### Criteria for treatment evaluation:

Treatment outcomes and prognosis^8^: 1) treatment outcomes: a. cure: the patient tested negative for *Burkholderia pseudomallei*, the imaging results were unremarkable, and the clinical symptoms disappeared completely after systematic treatment; b. improvement: the patient tested negative for *Burkholderia pseudomallei* despite the presence of residual lesions displayed by imaging equipment or clinical symptoms; c. stable condition: the patient was still positive for *Burkholderia pseudomallei* but his/her clinical symptoms were moderately alleviated or remained stable; d. death: the patient died of *Burkholderia pseudomallei* infection during the course of treatment. 2) Prognosis: a. free of recurrence/exacerbation: the patient was cured and remained negative for *Burkholderia pseudomallei* during the follow-up period without recurrence of any symptoms, or the patient showed an improvement or remained stable, i.e., negative for *Burkholderia pseudomallei* without exacerbation of symptoms; b. post-discharge mortality: the patient died of *Burkholderia pseudomallei* infection due to recurrence or aggravation of the condition.

### Statistical Analysis:

Medical records were imported into and sorted out via Excel 2019. Data analysis was performed using SPSS22.0. Measurement data following a normal distribution were expressed by “false±s” and comparison between two groups was examined by the t-test. Measurement data with a skewed distribution were represented by medians (interquartile range, IQR) and examined by the rank sum test. Antibiotic susceptibility testing was conducted using WHONET 5.6. Significance was set at the level of P <0.05. x̄

## RESULTS

The baseline characteristics of the 45 melioidosis cases showed that there was a higher proportion of male patients (84.44%) and suburban residents (64.44%); *Burkholderia pseudomallei* specimens were mainly derived from blood (46.67%) and sputum (26.67%); these patients tended to visited the departments of infection (44.44%) and respiratory medicine (35.56%) upon initial diagnosis. Table-I. In the 45 patients, melioidosis was mainly manifested by low fever (31.11%) and pulmonary infection (22.22%), while increased CRP (73.33%), #WBC (68.89%), and PCT (66.67%) were frequently seen in the results of auxiliary examinations. Table-II.

**Table-I:** Distribution of baseline characteristics of the 45 melioidosis patients [n(%)]1.

Baseline characteristics (n =45)		Percentage (%)
Sex	Male	38(84.44)
	Female	7(15.56)
Ethnic group	Han	40(88.89)
	Ethnic minorities	5(11.11)
Area of residence	Urban area	16(35.56)
	Suburb	29(64.44)
Specimen type	Blood	21(46.67)
	Sputum	12(26.67)
	Pericardial fluid	6(13.33)
	Pleural effusion	2(4.44)
	Urine	2(4.44)
	Pus	2(4.44)
Underlying condition(s)	Diabetes	7(15.56)
	Pulmonary tuberculosis	4(8.88)
	Chronic hepatitis	5(11.11)
	Hypertension	6(13.33)
	Hyperlipemia	7(15.56)
	Prostatic hyperplasia	2(4.44)
Department of initial diagnosis	Infection	20(44.44)
	Respiratory medicine	16(35.56)
	Endocrinology	4(8.89)
	Other departments	5(11.11)

**Table-II:** Clinical features and manifestations of the 45 melioidosis patients [n(%)]2.

Clinical features (n =45)		Percentage (%)
Clinical manifestations	Low fever (37.4-38°C)	14(31.11)
	Moderate fever (38.1-39°C)	2(4.44)
	Pulmonary infection	10(22.22)
	Urinary tract infection	3(6.67)
	Sepsis	3(6.67)
	Visceral abscess/purulent inflammation	4(8.89)
Auxiliary examinations	Increased #WBC	31(68.89)
	Increased CRP	33(73.33)
	Abnormal liver function	2(4.44)
	Abnormal kidney function	2(4.44)
	Water-electrolyte imbalance	3(6.67)
	Abnormal findings on lung imaging	7(15.56)
	Splenohepatomegalia (i.e., enlargement of spleen and liver) under ultrasound	2(4.44)
	Increased PCT	30(66.67)

Burkholderia pseudomallei strains were collected from the 45 patients respectively for drug resistance analysis, and the results showed that Burkholderia pseudomallei was highly sensitive to IPM (100%), MEM (24.44%), and CAZ(93%). Table-III. Of the 45 melioidosis patients, 23 (51.11%) were cured, and 21 showed an improvement (33.33%) or remained stable (13.33%); the post-discharge recurrence/exacerbation rates were relatively high (recurrence within 6 months: 4.44%; recurrence within a year: 6.67%); two cases of death were documented (in-hospital mortality: 2.22%; post-discharge mortality: 2.22%). Table-IV.

**Table-III:** Drug resistance rates of 45 strains of Burkholderia pseudomallei to antibiotics [n(%)]3.

Antibacterial	Total strains (n)	Susceptible strains (n)	Susceptibility rate (%)
SXT	45	40	88.89
CIP	45	40	88.89
CAZ	45	42	93.33
IPM	45	45	100.00
MEM	45	45	100.00

**Table-IV:** Analysis of treatment outcomes and prognosis of the 45 melioidosis patients [n(%)].4

Treatment outcomes and prognosis [n =45]		Percentage (%)
Treatment outcomes	Cure	23(51.11)
	Improvement	15(33.33)
	Stable condition	6(13.33)
	In-hospital mortality	1(2.22)
Prognosis	No recurrence/exacerbation within a year	39(86.67)
	Recurrence/exacerbation within 6 months	2(4.44)
	Recurrence/exacerbation within a year	3(6.67)
	Mortality at a year after discharge	1(2.22)

## DISCUSSION

Melioidosis is a typical amphixenosis of extremely high communicability and perniciousness as an endemic disease highly prevalent in tropical and subtropical regions such as Hainan Province and part of Guangdong Province in China.^9^-^11^ When melioidosis first occurs, patients have no distinct symptoms but malaise and low-grade fever resembling common diseases such as common cold or fever. As melioidosis progresses, the body temperature rises gradually in parallel with the emergence of symptoms of respiratory infection, which may result in death if left untreated.^12^-^14^ *Burkholderia pseudomallei* is a highly motile, saprophytic, environmental bacterium that extensively exists in water, soil, and air and can cause melioidosis in humans when damaged skin or mucous membrane lining of the respiratory tract is exposed to contaminated water, soil or air.^15^,^16^ Antibiotic therapy is currently the mainstay of clinical treatment for melioidosis patients, which has remarkably improved the survival rate and prognosis of melioidosis patients. However, treatment outcomes are strongly affected by the resistance of *Burkholderia pseudomallei* to antibacterials.^17^-^19^ In patients infected with *Burkholderia pseudomallei*, the pathogenic bacterium invades tissues and blood and causes death of normal cells and tissues by constant reproduction in the body, thereby contributing to dysfunction and inflammatory response of tissues and organs. Besides, long-term use of antibiotics is likely to induce pathogenic variation of *Burkholderia pseudomallei*, and improper use of antibacterials may adversely affect the treatment outcomes.^20^-^22^ In this study, the clinical features, manifestations and drug resistance of melioidosis were analyzed to provide a reference for clinical prevention and control of the disease.

The study results showed that among the 45 melioidosis patients, males and people living in suburbs outnumbered females and urban residents, respectively (male vs female: 84.44% vs 15.56%; urban area vs suburban: 64.44% vs 35.56%), conforming to the study by Fairley L et al.^23^ All this suggests that males and suburban residents are susceptible to *Burkholderia pseudomallei* infection. Additionally, 45 strains of *Burkholderia pseudomallei* were basically derived from patients’ blood and sputum (blood: 46.67%; sputum: 26.67%). This was consistent with the study by Ali M et al.^24^, offering positive evidence for the identification of damaged skin and respiratory tract as major channels of *Burkholderia pseudomallei* infection. The distribution of clinical features and manifestations showed that melioidosis was mainly manifested by low fever (31.11%) and pulmonary infection (22.22%), and serology test results indicated increased in CRP (73.33%), #WBC (68.89%), and PCT (66.67%). These findings were in agreement with the study by Rao C et al.,^25^ demonstrating the reliability of clinical screening and diagnosis of melioidosis based on such signs and symptoms and serology tests to improve the accuracy of early diagnosis. The antibiotic susceptibility test results indicated that Burkholderia pseudomallei was highly sensitive to IPM, MEM and CAZ, with the drug sensitive rates of 100%, 100%, 93.33% respectively.

### Limitations of the study:

In clinical practice, antibiotic susceptibility testing may facilitate the choice of antibacterials to improve the treatment outcomes and prognosis of melioidosis patients. It should be noted that this study has a small sample size and simple experimental data as it is constrained by a lack of human resources, funding and time. These limitations may bring a bias to the study results. The research value is expected to be expanded in the future.

## CONCLUSION

Low fever, pulmonary infection, and increases in serum inflammatory markers are major clinical features of melioidosis. *Burkholderia pseudomallei* presents high sensitive rates to antibacterials such as IPM, MEM, CAZ and SXT, and thus in clinical practice, the choice of antibacterials should be made by reference to susceptibility test results.

### Authors’ Contributions:

**HH & YS:** Designed this study, prepared this manuscript, are responsible and accountable for the accuracy and integrity of the work.

**XH & GZ:** Collected and analyzed clinical data.

**SW & XW:** Data analysis, Significantly revised this manuscript.
